# Automated control of end-tidal volatile anaesthetic concentration using the MIRUS™ system: a comparison of isoflurane, sevoflurane and desflurane in anaesthesia

**DOI:** 10.1186/cc14575

**Published:** 2015-03-16

**Authors:** V Vinnikov, D Drees, J Herzog-Niescery, P Gude, H Vogelsang, B Cevik, T Weber, M Bellgardt

**Affiliations:** 1 St. Josef-Hospital, Ruhr-Universität, Bochum, Germany

## Introduction

The new MIRUS™ system as well as the established AnaConDa® system uses a reflector to conserve volatile anaesthetics (VA) [1]. Both systems act with commercially available ICU ventilators. In contrast to AnaConDa®, MIRUS™ includes an automated control of end-tidal VA concentrations. In this study we compared feasibility, costs and recovery times after anaesthesia with isoflurane (ISO), sevoflurane (SEVO) or desflurane (DES) in ventilated and spontaneously breathing patients.

## Methods

The study was approved by the appropriate institutional review board. After written informed consent, 63 ASA I to III patients undergoing elective hip or knee replacement surgery under general anaesthesia were included. Patients were randomly organised into three groups (20 to 22 each). Anaesthesia was induced with intravenous anaesthetics. After tracheal intubation MIRUS™ automatically adjusted the end-tidal VA concentration to 1.0 MAC. Patients were ventilated with the Puritan Bennett 840 ICU ventilator. After 1 hour of anaesthesia with 1.0 MAC the ventilator mode was switched from SIMV VC+ (totally controlled ventilation, passive patient, with a tidal volume of 8 ml/IBW) to proportional assist ventilation with 50% support (active patient). At the end of surgery the MIRUS™ system was stopped (MAC set to 0.0) and recovery times were measured.

## Results

Patients were comparable in age, height, weight and operation time. In 60/63 patients a MAC of 1.0 was reached by MIRUS™. Therefore, ISO 11.2 ± 3.3 ml/hour, SEVO 24.3 ± 4.8 ml/hour or DES 41.7 ± 7.9 ml/ hour (mean ± SD; *t *test: *P *< 0.001) were used during passive ventilation. During patients' active ventilation, mean VA consumptions of ISO 9.6 ± 5.1 ml/hour, SEVO 19.4 ± 9.6 ml/hour or DES 35.5 ± 23.0 ml/hour were detected (NS between passive and active patients). ISO was the cheapest VA (€2.70 ± 3.10/hour passive patient, €1.90 ± 2.30 active patient), followed by SEVO (€8.40 ± 3.70 passive patient and €6.8 ± 3.8 active patient) and DES (€9.6 ± 4.1 passive patient and €8.6 ± 6.5 active patient). Recovery times were significantly shorter after SEVO and DES compared with ISO (minutes:seconds; ISO 9:31 ± 6:04, SEVO 6:19 ± 2:56, DES 5:27 ± 1:59).

## Conclusion

This study showed that MIRUS™ could automatically control end-tidal VA concentrations in ventilated and spontaneously breathing patients. Using ISO reduces costs. Further studies must be taken to analyse feasibility, costs and recovery times of ISO, SEVO and DES used for sedation in an ICU setting.
